# An Ecodevelopmental Framework for Engaging Diverse Youth in Foster Care and Their Families Into Technology-Based Family Intervention Research Trials

**DOI:** 10.3389/fdgth.2022.866139

**Published:** 2022-05-13

**Authors:** Johanna B. Folk, Heman Gill, Catalina Ordorica, Christopher A. Rodriguez, Evan D. Holloway, Jocelyn Meza, Marina Tolou-Shams

**Affiliations:** ^1^Department of Psychiatry and Behavioral Sciences, University of California, San Francisco, San Francisco, CA, United States; ^2^Department of Psychiatry and Biobehavioral Sciences, University of California, Los Angeles, Los Angeles, CA, United States

**Keywords:** child welfare, clinical trial, foster care, social environment (MeSH), telehealth, patient engagement

## Abstract

Family-based interventions delivered via telehealth are a promising mode for overcoming barriers to behavioral health treatment among youth in foster care and their families. There is a dearth of research, however, regarding effectiveness of these interventions for youth in foster care, who commonly exhibit complex behavioral health treatment needs. Clinical research in this area directly relates to equity in service access and quality for these youth and families, with numerous barriers and enabling factors to consider in order to improve engagement in clinical trials and bolster the evidence base. We present a framework to better understand the multi-systemic factors impacting youth and family engagement in clinical research on family-based telehealth interventions, drawing on relevant theory, including the bioecological model and ecodevelopmental theory. We also draw on our experiences conducting technology-based clinical research through the Family Telehealth Project, an evaluation of a brief family-based affect management intervention designed specifically for youth in foster care and their families, as a case example. Recommendations for promoting engagement in clinical research on family-based telehealth interventions with diverse youth in foster care and their families are provided.

## Introduction

The Child Welfare System in the United States [sometimes referred to as the Family Regulation System (1)] is charged with investigating reports of abuse or neglect and intervening to protect children, as needed; interventions may include mandating family-based services and placing children into foster care. In 2020, 407,493 youth ages 0 to 20 years were removed from their family and placed into foster care (2). Indigenous and Black youth are at highest risk of foster care placement before age 18 (3); these inequities are driven by systemic factors (i.e., structural racism), not the commonly noted spurious “risk factor” of race (4). Youth in foster care commonly exhibit significant behavioral health treatment needs, including histories of complex trauma, mental health symptoms, and substance use. Family-based interventions are a gold-standard for youth in foster care, having demonstrated efficacy in improving mental health, substance use, educational, and delinquency outcomes (5, 6). However, geographical distance between youth in foster care and their families of origin can impede participation in family-based interventions, both in clinical practice and research trials (7, 8).

Telehealth is one promising mode for overcoming barriers (e.g., transportation) to accessing family-based treatment with youth in foster care (9, 10), however, further research regarding the effectiveness of family-based interventions delivered via telehealth is needed. Most research on telehealth service delivery has been with individual behavioral health interventions and less complex clinical presentations than commonly found among youth in foster care (11, 12). Youth in foster care are also disproportionately impacted by the digital divide, with restricted access to technology compared to their peers (13–15), which can be a barrier to participation in technology-based interventions and clinical research. As such, to maximize engagement (e.g., enrollment, retention) in clinical research on family-based telehealth interventions with youth in foster care and their families, it is crucial to attend to barriers and enabling factors specific to both system involvement and technology.

**Figure 1:**
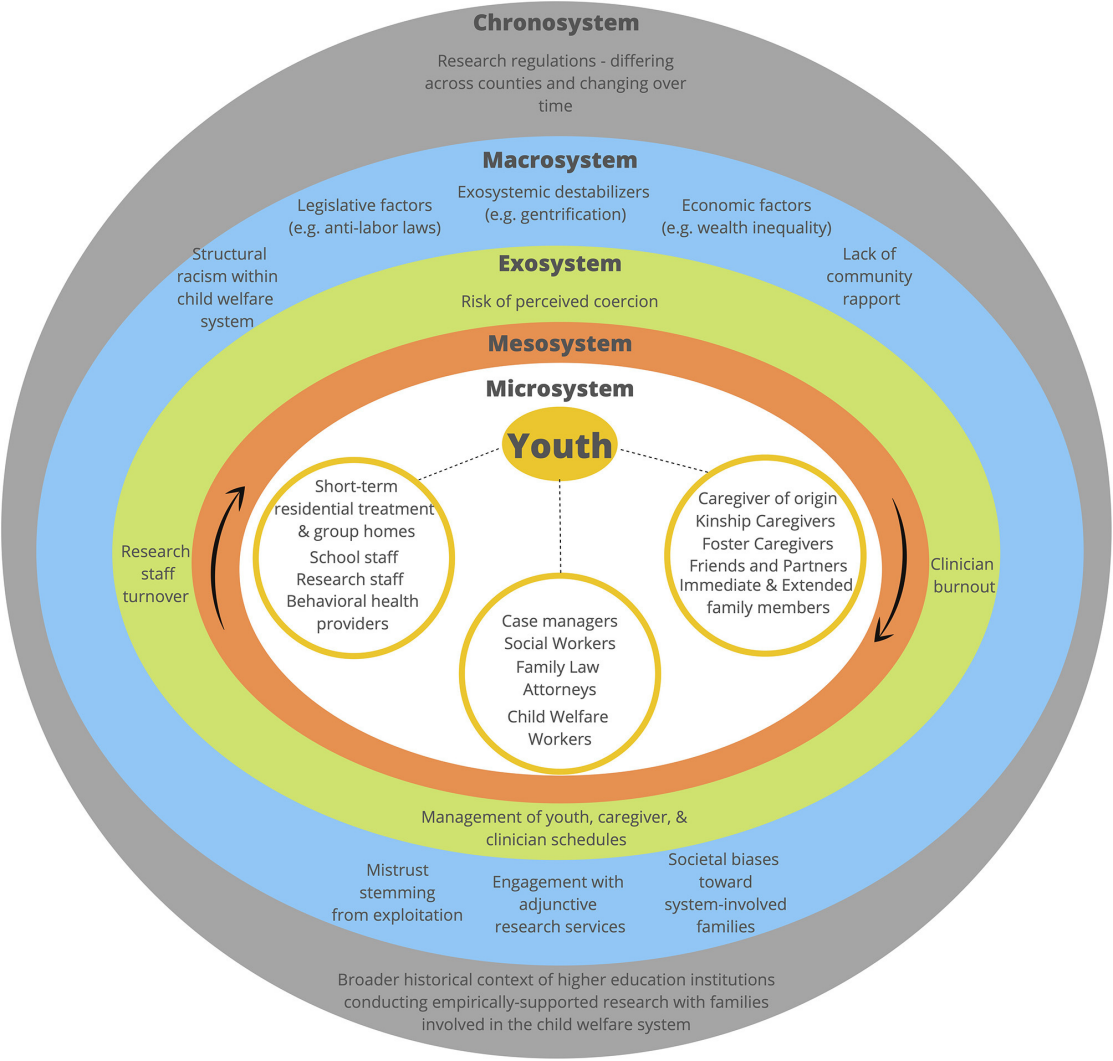
An ecodevelopmental framework for understanding and enhancing engagement in clinical research on family-based telehealth interventions.

Toward this aim, we present a framework for conceptualizing factors impacting engagement in research evaluating family-based telehealth interventions while youth are in foster care (see Figure 1). The authors draw on the bioecological model (16) and ecodevelopmental theory (17), as well as experiences conducting technology-based clinical research with youth in foster care and their families through the Family Telehealth Project. We offer concrete suggestions for overcoming barriers and promoting enabling factors to engagement in order to advance the field of digital health equity research with underserved youth and families.

## An Ecodevelopmental Framework for Understanding and Enhancing Engagement in Clinical Research on Family-Based Telehealth Interventions

Ecodevelopmental Theory (17) extends Bronfenbrenner's bioecological model of human development (16) (i.e., micro-, meso-, exo-, macro-, and chrono-system influences on development and behavior) by accounting for the role of different contexts and developmental processes. Ecodevelopmental theory is particularly relevant to understanding engagement in clinical research on family-based interventions while youth are in foster care since such system involvement occurs during key developmental periods, from childbirth through adolescence. In addition to genetic and hormonal influences *in utero* from biological parents which directly influence early childhood development (e.g., attachment, temperament), the family microsystem has a fundamental influence on youth behavior due to prolonged and frequent interactions with family members. The family microsystem also reciprocally influences peer and romantic partner microsystems, institutional systems at the meso-system level (e.g., schools), and youth-family interactions reciprocally interact within proximal contexts (e.g., peer, community, cultural).

Factors affecting engagement (e.g., barriers and enabling factors) of youth in foster care and their families in clinical research should not be understood as uniform or static but rather as dynamic and contextual (see Figure 1). At the individual level, youth in foster care experience elevated behavioral health treatment needs, often due to sequelae of complex trauma. They also have complex micro-systems (i.e., direct interpersonal influences) consisting of not only family, friends, and partners, but also kinship or foster caregivers, child welfare workers, case managers, family law attorneys, school staff, and behavioral health clinicians, among others. The microsystem includes both physical and virtual (18) relationships; the physical microsystem consists of activities, social roles, and interpersonal relations in face-to-face (in-person) settings, whereas the virtual microsystem involves these same features on a digital platform. Individuals within the micro-system have the most direct and frequent contact with youth; researchers must therefore sustain collaborative relationships, build rapport, and maintain consistent communication with multiple persons to facilitate engagement in clinical intervention research for this specific population.

The meso-system involves interactive influences between various micro-systems (17) (e.g., parent/caregiver of origin and child welfare staff, family law attorneys and behavioral health clinicians, foster family and service delivery systems). To promote engagement at the meso-system level, researchers must assess the strength of existing relationships and encourage collaboration between individuals and systems that may not interact regularly or effectively with one another, such as child welfare workers and caregivers of origin (19).

Exo-systemic factors (i.e., indirect, interactive influences) (17) directly influence successful clinical research, including management of youth's, caregivers', and/or clinicians' schedules when coordinating family-based intervention sessions, clinician burnout, clinician and research staff turn-over, and research and legal regulatory requirements specific to this population. Specific to telehealth intervention trials, access to technology and privacy for participation in sessions and assessments may be impacted by youth's placement (e.g., youth in group homes may be sharing devices or have rules around unsupervised use of devices needed for session participation). Further, families face multiple pressures from the child welfare system to comply with mandated reunification plans, increasing the risk of coercion to participate in clinical intervention research. Families may perceive clinical intervention research participation to be required or believe participation will look favorable to the child welfare system. Although researchers have limited control/influence on exo-systemic factors and their effects on youth and families, researchers must be responsive to them to maximize youth and family engagement, particularly when it comes to perceived coercion to participate in research.

At the broader macro-systemic level, shifting societal factors that increase the likelihood of child welfare involvement should be considered to conduct prevention and intervention research more successfully. Researchers must be aware of, and responsive to, contributors to disproportionate system involvement of ethnoracial minoritized groups (3), including discrimination, structural oppression [e.g., increasingly expansive surveillance and net-widening (20), historical and ongoing effects of structural racism (4, 21)], as well as legislative and economic factors [e.g., family income, county-level poverty, and county-level income inequality (22, 23)] that contribute to child welfare system involvement, as they change over time. Macro-level factors affecting willingness to participate in clinical research may include a lack of trust toward the child welfare and affiliated healthcare systems (24–26) stemming from exploitative practices, systemic racism, and societal stereotypes and biases held toward marginalized youth impacted by the child welfare system and their families (27). Further, many youth are also dually involved in the juvenile delinquency and dependency court systems, which can result in “falling through the cracks” in timely access to needed care given challenges to information-sharing and cross-system collaboration (28); Black and Latinx youth in the child welfare system are at particularly high risk of entering the juvenile justice system (29). Specific to telehealth interventions, factors such as changes in access to technology (e.g., increasing internet access in rural areas) and wide-scale delivery of telehealth interventions which may influence their acceptability (e.g., during the COVID-19 pandemic with restrictions to in-person behavioral health services) may impact engagement in research (30). Understanding the effects of such macro-level factors can help researchers develop intervention content informed by various social, political, and cultural influences on a youth's development before and while they are enrolled in clinical research, which may facilitate engagement.

The chrono-level (i.e., temporal influences) accounts for changes that occur within and between systems at the micro-, meso-, exo- and macro-systemic levels over time (17). Interactions between multiple systems and their effects on youth and families impacted by the child welfare system are dynamic, especially those influenced by temporal changes within the family microsystem (e.g., socioeconomic mobility) and societal influences at the macro-systemic level (e.g., shifting political climate, funding priorities for services and research). Based on our own experiences, bureaucratic delays and evolving requirements when obtaining required approvals to conduct research with youth and families involved in the dependency and delinquency courts, historical contexts between research institutions and the specific populations they serve, and inequities within the higher educational system, all contribute to temporal changes impacting clinical research. Researchers must continuously identify and address gaps in the implementation of evidence-based practice, recognize how our institutions contribute to challenges engaging families impacted by the child welfare system into research and expand clinical intervention research to historically underrepresented populations.

## The Family Telehealth Project

The authors draw on experiences conducting research through the Family Telehealth Project, which aims to improve behavioral health outcomes and reduce housing instability among youth in foster care (ages 12–18 years) through a family-based affect management intervention. Phase 1 involved the iterative adaptation of an empirically supported in-person family-based affect management intervention (31) for telehealth delivery and to meet the unique needs of adolescents impacted by the child welfare system and their caregivers of origin. The family-based affect management intervention being adapted was developed using the Social-Personal Framework (32), which recognizes adolescence is a period of significant emotional, cognitive, and physical changes. The Social-Personal Framework considers the interplay between individual, social, and environmental influences on adolescent risk, including individual factors, family context, and peer/partner influences. It has shown particular utility for understanding risk among adolescents in clinical settings and on probation (33, 34). The intervention includes an engagement session using motivational interviewing principles, four core modules, and a booster session (approximately 11 h of intervention time total). Core session content includes affect management, parental monitoring, and communication skills. Two clinicians work with each family to meet individually with the youth and the caregiver and then co-lead the family session. Family sessions allow for shared skill-building, practice, and discussion. Adaptation was conducted iteratively in collaboration with key stakeholders (youth, caregivers, child welfare supervisors, probation officers, judges, attorneys, school wellness staff) and through an open trial of the intervention. A caregiver-only version of the intervention is also available, covering the same core content and structure, when youth are unable to or not interested in participating. Phase 2 of the study involves an ongoing clinical trial to evaluate the effectiveness of the intervention.

## Strategies for Improving Engagement of Youth in Foster Care and their Families in Clinical Research

Researchers must actively consider the dynamic, multiple levels of influence on youth in foster care and their families when conducting clinical research on family-based telehealth interventions. This includes exploration of structural vulnerabilities and social determinants of health for minoritized youth impacted by the child welfare system. Using a justice, equity, diversity and inclusion framework can enable real-word exploration of structural vulnerabilities for minoritized youth impacted by the child welfare system and facilitate the development of strategies on how to disrupt these vulnerabilities; such strategies include examination of social determinants of health and the integration of community based participatory research. We propose key recommendations addressing each system level, that we hope will disrupt these vulnerabilities (see Table 1). These proposed strategies are not exhaustive and focus on considerations unique to youth in foster care and their families; we do not include standard best practices for clinical research applicable to general populations, though these certainly still apply. We highlight select recommendations using the Family Telehealth Project as a case example.

**Table 1:** Select recommendations for conducting clinical research on family-based telehealth interventions with youth in foster care and their families.

**Research Phase**	**Specific issue for consideration**	**Relevant systems and recommendations**
Preparation	**Community partnerships:** •Stakeholders (e.g., child welfare, behavioral health, attorneys) have vested interest and involvement in improving outcomes for system-impacted youth and families. •Sustainable community partnerships are integral to successful clinical intervention research.	**Mesosystem:** •Identify key stakeholders from existing university partnerships, federal, state-wide, and local databases, and the youth and families directly. •Incorporate stakeholders as key collaborators in all stages of the research process, ideally from the generation of an unmet clinical need through data analysis and dissemination of findings. •Ensure relevant stakeholders understand the overall goals of the intervention, referral process, and how systems considerations (e.g., family reunification plans, supervision of contact) are being addressed. •Identify ways to promote intervention sustainability after research funding ends. •Incorporate youth, family, and other relevant stakeholder perspectives into iterative adaptation and design of interventions to ensure the approach meets local needs, is acceptable, and is feasible to implement. •When permissible, equitably compensate stakeholders for their time contributing to the research process. When stakeholders cannot accept financial compensation, provide refreshments as a gesture of gratitude for donating their time and expertise.
	**Regulatory approvals:** •Some counties require court petitions and/or county behavioral health approval, in addition to university IRB approval.	**Macrosystem:** •Spend ample time before study begins researching county-specific requirements for conducting clinical intervention research with system-impacted youth. •If recruiting from multiple counties, create a tracking log of all the counties of interest for recruiting families and their requirements ahead of time. •Ensure university IRB approval is obtained with sufficient time to submit alongside the required court petitions (e.g., several months in advance). •For counties requiring attorney approval to approach youth for informed consent, create attorney consent forms, a visual guide to explain study procedures, and a spreadsheet to track attorney contact information. •Initiate a conversation with each county about the “rights” to all data collected. A research team may need to draft a memorandum of understanding (MOU) or exemption form based on the county's expectations and unique history of data use in research.
	**Study clinicians:** •For brief interventions, partnering with community-based clinicians to deliver study interventions can reduce the number of external providers involved in a youth's care, promote long-term sustainability of the intervention, and increase likelihood stakeholders will make study referrals. •Community-based clinicians have varying experience with manualized interventions and maintaining fidelity in clinical research trials, and numerous competing demands from their primary professional role.	**Exosystem:** •Create detailed workflows outlining research protocols relevant to clinicians (e.g., checklist of steps to prepare for a session); record training to facilitate onboarding of new clinicians and allow access to refresher material. •Provide training in flexible delivery of manualized interventions, including balancing of flexibility and fidelity in approach, and any empirically supported approaches necessary to deliver them (e.g., motivational interviewing). •Provide training in use of technology for intervention delivery and ensure clinicians' feel comfortable using any special features (e.g., screen sharing). **Macrosystem:** •Partner with supervisors to support community-based clinicians in incorporating the intervention into their standard care, including outside the research trial if there is already evidence to support the intervention's effectiveness. •Identify whether and how interventions can be billed as part of clinical services when delivered by community-based clinicians to promote long-term use.
Consent, Engagement, and Retention	**Caregiver Consent:** •Caregiver consent for youth to participate in research is often required, however caregivers in system-impacted families may have had their parental rights terminated. •Caregivers may believe participation in clinical research will impact their ongoing dependency case.	**Microsystem:** •During initial eligibility screening, ask caregivers if their parental rights have been terminated for the referred youth. If so, identify legal signing guardian (e.g., supervising social worker, family court presiding judge) prior to consent appointment. •Ensure consent process and recruitment materials make clear that participation in the clinical research trial will not impact their ongoing dependency case or decisions about reunification plan. **Exosystem:** •When appropriate, obtain a waiver of parental consent from the university IRB so youth can consent to research without caregiver consent (i.e., youth 12 years+)
	**Coordination with out-of-home placements:** •Youth in foster care are separated from their caregiver and may be without access to a personal form of communication. •Out-of-home placements have varying restrictions on technology use, including for therapeutic purposes with outside clinicians.	**Mesosystem:** •Coordinate research and intervention appointments with the caregiver and the youth's placement (e.g., group home, short-term residential treatment program) to ensure youth (and caregiver, when relevant) are present. Coordination with social workers and attorneys may also be necessary for screening and consent appointments. •Communicate with out-of-home placements to coordinate availability of a private space and necessary technology prior to the first appointment and send appointment reminders.
	**Clinical Intervention Accessibility:** •System-impacted youth and families have diverse linguistic, cultural, and accessibility needs. •System-impacted youth and families are often separated, preventing access to family-based interventions.	**Microsystem:** •Conduct pre-intervention session with participants focused on enhancing engagement using motivational interviewing principles and troubleshooting possible barriers to session attendance and participation. •Collect data on cultural relevance/acceptability of intervention and adapt iteratively if indicated. **Exosystem:** •Hire enthnoculturally diverse and bilingual/multilingual staff and clinicians. •Budget for translation and/or interpretation services in grants. **Macrosystem:** •Ensure accessibility of intervention materials to youth and families with: •Varying visual and auditory abilities (e.g., verbal discussion of intervention materials, enable auto- and/or live closed captioning during telehealth sessions). •Different linguistic (e.g., Spanish, Arabic, Hmong) and/or cultural (e.g., Latinx) backgrounds.
	**Technology Accessibility:** •Youth in foster care are disproportionately impacted by the digital divide and may not have access to necessary technology to participate in sessions. •Technology literacy can vary for both youth and caregivers.	**Microsystem:** •Develop a standardized set of questions to assess technology access (e.g., what devices youth and family have available, Wi-Fi access and stability of connection) and privacy considerations prior to beginning clinical intervention sessions; consider providing devices and funds for data plan costs to promote participation, as well as headphones to promote privacy. •Provide instructional resources on how to use technology platforms, in both written and visual (e.g., video) formats and in multiple languages; provide personal tutorials to families, as needed. **Macrosystem:** •Ensure handouts and videos are viewable on small screens (e.g., phone) so large device access is not required to participate.
Intervention	**Clinical Intervention Relevance:** •Youth in foster care experience elevated behavioral health needs (e.g., mental health, substance use), often resulting from complex trauma. •Placement out-of-home disrupts familial relationships, which may have already been strained.	**Individual:** •Ensure content is trauma-responsive (e.g., providing psychoeducation about the impact of trauma on development). •Focus skill-building on transdiagnostic areas such as emotion regulation; teach skills to both you and caregivers. **Microsystem:** •Address maintenance of family connections (when appropriate) within imposed limitations by child welfare system; incorporate communication skill building to improve family relationships.

Interdisciplinary collaboration with key stakeholders working with youth and families impacted by the child welfare system is critical, starting from the preparation stage. In the Family Telehealth Project, we regularly consulted stakeholders from the child welfare, behavioral health, and legal (e.g., attorneys, family court judges) systems on research procedures (e.g., referral sources) and intervention content adaptation. Stakeholder input was gathered through formal data collection (i.e., focus groups) and informal meetings. For example, stakeholders reviewed all intervention session scripts in detail with the study team during monthly meetings in Phase 1 of the project. Stakeholders provided feedback on relevance of session content (e.g., maintain emphasis on substance use and sexual and reproductive health, addition of psychoeducational content on the impact of trauma and topics like dating violence) and suggested considerations for delivering content while youth are separated from their caregivers (e.g., how caregivers can engage in parental monitoring from a distance and within the context of supervised contact). Stakeholders also informed modification of activities to telehealth, noting considerations around length of sessions based on their experiences with youth over telehealth. Youth and caregivers also provided feedback on intervention content through qualitative interviews and session feedback forms during Phase 1; youth and caregivers provided invaluable insights into the utility of skills taught during sessions and overall acceptability of the intervention.

Regulatory approvals are complex when conducting research with system-impacted youth and families. In addition to approval by the institutional review board, many counties require court orders and county mental health approval to recruit youth and their caregivers into clinical intervention studies. In our experience, instructions for obtaining necessary approvals are often not clearly documented and it can require significant time and resources to determine requirements and navigate the approval process. In the Family Telehealth Project, we obtained approval to recruit for our clinical research study in five California counties; three required court orders, four required county mental health approval, and one had no formal requirements for research. We were unable to obtain approval in three additional counties due to lack of resources in the court to review and approve research proposals or requirements that the county own the data collected (rather than the researcher). Each county's approval process was unique and, in some cases, required research staff to draft court orders themselves, a skill not commonly required of research assistants in academic medicine. Each county also had unique requirements for conducting the research itself; for example, one required attorneys to provide consent prior to research staff contacting youth to explain the study and screen them for eligibility; unique attorney permission forms had to be created along with materials for attorneys to understand the research and permission process. This process was incredibly time and labor intensive. Researchers should therefore budget ample time before beginning a study researching county-specific requirements and obtaining necessary approvals. However, such system-level barriers require system-level solutions. Given the clear impact of clinical research on equity in services access and quality for youth in foster care and their families, counties should prioritize developing clear and standardized processes to support researchers dedicated to improving the health of system impacted youth and their families.

Accessibility of the intervention, both for clinicians to deliver and for youth and families to engage with, is multi-faceted. In the Family Telehealth Project, we trained clinicians at a community-based agency and a short-term residential treatment program to deliver the intervention in collaboration with study clinicians. We did so to promote continuity of care for youth already in a therapeutic relationship, ensure sustainability of the intervention after research funding ends, and build capacity among community clinicians in delivering manualized, skills-based interventions. Creation of detailed workflows and protocols related to session delivery, as well as providing training in flexible manualized intervention delivery and use of technology was critical. Community clinicians all had to complete human subjects training and be added to the institutional review board application. Clinician turnover was a major impediment; in one agency, all of the trained clinicians left their position within a year and 85% of those trained never had/referred an eligible client. Clinicians with potentially eligible clients should be carefully selected, and trainings held as close to enrollment of a potential participant as possible to ensure retention and application of knowledge. Further, training clinical supervisors in the intervention and recording training sessions for later review, can help ensure knowledge is retained within an agency even if individual clinicians leave.

For youth and caregivers, we had to account for technology factors (e.g., phone vs. computer use), varying visual and auditory abilities, and cultural and linguistic backgrounds, in delivery of the intervention. We found tailoring sessions for accessibility on a phone most useful, as many families did not have a larger screen device (e.g., computer, tablet) for participation. For example, handouts were designed for readability on a smaller phone screen. We did not provide devices or internet access to families in our study, though these are other ways to facilitate access to necessary materials for participation. For youth in group home placements, coordination with clinical staff on-site was instrumental to ensuring youth had device access and privacy to participate. Other accessibility accommodations included use of closed captioning and audio for all videos in the intervention, available in both English and Spanish; live closed captioning on videoconferencing and the chat could also be used during intervention sessions for participants with limited hearing abilities. We also hired multilingual/bilingual and ethnoculturally diverse staff to deliver the intervention and conduct research procedures with Spanish-speaking families; further, we are currently culturally adapting the intervention for Latinx families.

## Discussion

The proposed framework and recommendations are intended to guide researchers committed to promoting equitable delivery of evidence-based behavioral healthcare for diverse youth in foster care and their families. Although the COVID-19 pandemic and restrictions to in-person behavioral health services highlighted the need for empirically supported interventions delivered via telehealth, behavioral healthcare via telehealth could reduce access barriers for families in non-pandemic times as well (e.g., overcoming challenges related to distance and transportation). Clinical research in this area is crucial as it directly relates to equity in services access and quality for youth in foster care and their families. It is crucial we identify ways to overcome barriers to delivery of empirically supported behavioral health treatment for youth in foster care and their families; failure to do so perpetuates service access inequities and engagement for racial/ethnic minoritized youth, who are disproportionately represented among youth in foster care (20, 35). Through cognizance of the multiple people, systems, and influences at play in a youth's life who is impacted by the child welfare system, we hope the collective research community can work to advance knowledge and implementation of empirically supported behavioral healthcare that is feasible, acceptable, and culturally relevant to reduce inequities for this highly marginalized group of youth and families.

## Data Availability Statement

The original contributions presented in the study are included in the article/supplementary material, further inquiries can be directed to the corresponding author.

## Author Contributions

All authors participated in the conceptualization of the proposed ecodevelopmental framework, writing, and editing process. All authors contributed to the article and approved the submitted version.

## Funding

This work was supported by a gift from the Visa Foundation (PI: MT-S) and a grant from the American Psychological Association (PIs: JF and MT-S). Authors also received salary support for this work from the National Institute on Drug Abuse (K23DA050798, K24DA046569, and T32DA007250) and the National Institute on Mental Health (T32MH018261).

## Conflict of Interest

The authors declare that the research was conducted in the absence of any commercial or financial relationships that could be construed as a potential conflict of interest.

## Publisher's Note

All claims expressed in this article are solely those of the authors and do not necessarily represent those of their affiliated organizations, or those of the publisher, the editors and the reviewers. Any product that may be evaluated in this article, or claim that may be made by its manufacturer, is not guaranteed or endorsed by the publisher.
